# Fifteen Years' Experience of Infectious Diseases in a Public School
1Read at the May Meeting of the Bristol Medico-Chirurgical Society.


**Published:** 1897-09

**Authors:** W. Johnstone Fyffe

**Affiliations:** Physician to Clifton College


*Throughout Dr. Clarke's paper in the June number, pp.* 148—151, *read* "Staphylococcus" *for* "Streptococcus."

The Bristol

Medico-Chirurgical Journal.

SEPTEMBER, 1897.

### FIFTEEN YEARS' EXPERIENCE OF INFECTIOUS DISEASES IN A PUBLIC SCHOOL.[1]

BY

W. JOHNSTONE FYFFE, B.A., M.D. Dub.,

*Physician to Clifton College.*

INFECTIOUS diseases are the plague of schools. Their introduction into the school community gives rise to serious risks, and to great inconvenience. The prevalence of any one infectious disease in a school disturbs the educational work, interferes with games or with matches with other schools, and if the disease be of a severe or dangerous character, it may damage the sanitary reputation of the school so badly that it may require years to recover from it; and even when there is no cause for alarm or anxiety, or danger to life, an attack often means a period of prolonged ill health to boys, causes constant worry and vexation to parents and masters alike, and is a continual anxiety to the school doctor; consequently the duty of keeping watch and ward against the admission of infectious diseases into the community over which he exercises medical supervision becomes one of his most important functions, and especially does this duty require his vigilance at the commencement of each school term, for in spite of the regulations[2] prepared with much care and issued to

[1] Read at the May Meeting of the Bristol Medico-Chirurgical Society.
[2] Those in force at Clifton College are printed on the next two pages.

### CLIFTON COLLEGE.

### RULES FOR THE PREVENTION OF THE SPREAD OF INFECTIOUS DISEASES.

1.—The following diseases are considered as infectious:—Whooping Cough, Measles, German Measles or Roseola, Scarlet Fever, Typhus Fever, Enteric or Typhoid Fever, Small Pox, Chicken Pox, Diphtheria, Influenza, Mumps, Ringworm, Purulent Ophthalmia.

2.—No boy shall enter or return to the College who has had any infectious disease within a month, or who has been exposed to infection, without his Parents or Guardian giving notice to the Head Master and obtaining his permission. The required form of notice (A) can be had on applying to the Secretary.

3.—No boy shall enter or return to the College from a HOUSE in which there has been any infectious disease, or any person convalescent from such disease, until the following conditions have been complied with:—

(1) The House must be thoroughly disinfected in cases of Scarlet Fever, Typhus Fever, Typhoid or Enteric Fever, Small Pox or Diphtheria.

(2) Notice must be given to the Head Master and his permission obtained previous to the boy's return. The required form of notice (B) in the case of a boy having left the affected House; (C) in the case of the person affected having been removed from the House; (D) in the case of the boy having remained in the same House as the person affected, can be had on applying to the Secretary.

4.—CLOTHES worn during sickness or convalescence by a boy having had any infectious disease must not be sent with him when he returns to School. This Rule applies to all books and papers which he may have had with him during his illness or convalescence, and to boxes and portmanteaus which may have been in his room.

5.—A boy who has been exposed to infection from any of the above-named diseases during vacation time, *but who has previously had the disease in question*, may return at once to School, provided he comes direct to the Sanatorium for disinfection before entering his Master's House.

### SPECIAL RULES REGARDING ENFORCED ABSENCE ON ACCOUNT OF INFECTIOUS DISEASE.

Case A.—A boy who has been ill with—

Whooping Cough, may return to College in six weeks from the commencement of whooping, if the cough has ceased.
Measles „ „ in three weeks from the commencement of the illness, if all cough has ceased.
German Measles or Roseola „ „ in three weeks from the commencement of the attack, if there is no desquamation.
Scarlet Fever „ „ in not less than six weeks from the date of appearance of the rash, if all desquamation has ceased.

| | | | |
|---|---|---|---|
| TYPHUS FEVER, | may return | to College | when strength is sufficiently restored; average time six weeks to two months. |
| *TYPHOID or *ENTERIC FEVER | ,, | ,, | when strength is sufficiently restored; average time about two months. |
| SMALL POX | ,, | ,, | when every scab has fallen off from the head and body: not under four weeks. |
| INFLUENZA | ,, | ,, | in fourteen days from the beginning of the illness. |
| DIPHTHERIA | ,, | ,, | in not less than four weeks from the beginning; when strength is restored. |
| CHICKEN POX | ,, | ,, | when every scab has dropped off; two to three weeks. |
| MUMPS | ,, | ,, | in twenty-four days from the commencement; if all glandular swelling has subsided. |
| *RINGWORM | ,, | ,, | when all active diseased growth has ceased; and no patches or broken hairs can be discovered. |
| OPHTHALMIA | ,, | ,, | when the case is pronounced well by the Medical Attendant. |

* A Special Medical Certificate required.

CASE B—A boy who has not already had the disease in question, and who has been living in the same House with the affected person up to the day on which the disease declared itself, and then has been *immediately* removed to another House; or (CASE C), if the affected person has been immediately removed, must not return to College until the following time has elapsed after such removal has taken place:—

| | | | |
|---|---|---|---|
| WHOOPING COUGH ... ... ... ... ... ... | 16 days | SMALL POX ... ... ... ... ... ... | 21 days |
| MEASLES ... ... ... ... ... ... ... ... ... | 16 ,, | CHICKEN POX ... ... ... ... ... ... | 21 ,, |
| GERMAN MEASLES (ROSEOLA) ... ... | 21 ,, | DIPHTHERIA ... ... ... ... ... ... | 12 ,, |
| SCARLET FEVER ... ... ... ... ... ... | 14 ,, | MUMPS ... ... ... ... ... ... ... | 24 ,, |
| TYPHUS FEVER ... ... ... ... ... ... | 21 ,, | INFLUENZA ... ... ... ... ... ... | 14 ,, |
| ENTERIC (TYPHOID) FEVER ... ... ... | 21 ,, | | |

CASE D—A boy still living in the House where any of the above diseases break out, *and who has not already had the disease in question*, may return to School at the end of one fortnight after the last case has been declared free of infection by the Medical Attendant.

N.B.—Every boy who has been exposed to infection must come to the Sanatorium for disinfection before he goes to his Master's House.

Any sick boy may be sent to the Sanatorium, on the advice of the Medical Officer, or in a case of emergency by the House Master.

Day boys are admitted (if there be room) to the Sanatorium (for infectious illness) at a certain charge, and may be attended by their Family Doctor, unless the Parent should request the attendance of the College Doctor.

the parents or guardians of the boys, there is, I am sorry to say, a tendency occasionally to evade or escape from the rules laid down for their guidance.

I will give you an example. In the last term of 1896, Clifton College started with a clean bill of health. This continued for a fortnight, when a boy in one of the masters' houses developed mumps, and was at once removed to the sanatorium. On enquiring I found that this boy's brother at home had had a swelling at the side of his jaw. No one thought anything of it. It was not recognised as a case of mumps, and the Clifton boy was sent off to school. There can be little doubt that he had caught the disease from his brother, and the result of that mistake (which, of course, arose from ignorance) was, that 110 cases of mumps took place in the term, some of them being very severe. Probably if the medical attendant of the family had been called in the mistake would not have been made. In spite, therefore, of printed regulations, instances of this kind, arising from ignorance and occasionally although rarely from neglect in following the instructions, permit the introduction of some case of infectious disease which may spread with great rapidity through a young and very susceptible community.

The infectious diseases upon which I will venture to offer a few remarks to you are: influenza, sore throat, scarlet fever, measles, German measles, small-pox, chicken pox, diphtheria, whooping cough, mumps, ringworm, contagious impetigo, jaundice, and ophthalmia. The list is a long one, and therefore my remarks on each must be brief, and will touch mainly upon the points which have presented themselves to me during nearly fifteen years' experience as Physician to Clifton College.

**Influenza.**—We have had three visitations from this disease. One was in 1890, the details of which I brought before our local branch of the British Medical Association in the same year.[1] A second visitation occurred in 1895, when there were about 100 cases among the boarders. The type of the disease was severe, and sixteen cases of pneumonia occurred among them. Two of these ended in empyema. In both the chest was incised and drained by my colleague, Mr. Arthur Prichard. All these cases

[1] *Bristol M.-Chir. J.*, 1890, viii. 147.

recovered. A third case of bilateral empyema occurred in one of the town boys under my care. Both sides of the chest were opened by Dr. James Swain, and the boy quite recovered.[1]

A third visitation of influenza took place during the first term of the present year. There have been forty-four cases in all up to this date, very mild in character with one exception, but having this peculiarity, that in almost every case there was subacute inflammation (non-follicular) of the tonsils and arches of the palate. In the exceptional case I have just referred to, the throat symptoms were severe, but rapidly subsided, and were followed by the onset of pneumonia at the right base. Symptoms of pleuritic effusion set in, and in a fortnight after his admission seventy ounces of serum were aspirated from his right chest by Mr. Arthur Prichard. The case ended in empyema. The chest was incised and drained, but began to refill, when an accident occurred; about an inch or more of the drainage tube broke off inside the chest. Mr. Prichard then removed about an inch of the fifth rib, and succeeded in extracting the fragment of tube. Improvement immediately took place. The temperature fell almost to the normal point in the course of a few hours. After washing out the pleural cavity with warm boracic solution, the recovery was uninterrupted and rapid, and the patient went home quite convalescent.

**Sore Throat.**—Different forms of sore throat are by far the most common of the ailments affecting a public school. So far as I have observed, there are three special forms of sore throat to be met with among the boys. First, parenchymatous tonsillitis, generally affecting one tonsil, attended by pain and fever, occasionally ending in suppuration, and sometimes in a rheumatic attack, and probably best treated by the salicylates and alkalies. I have never regarded this form of sore throat as infectious, but some school doctors do regard it in that light—among them Dr. Haig Brown, the medical officer of Charterhouse. The second form of sore throat is follicular tonsillitis, a decidedly infectious disease, attended with yellowish or grey exudation on the tonsils, which exudation is, I believe, crowded with

[1] This case was reported in the *Bristol Medico-Chirurgical Journal* for December, 1893.

streptococci. Such cases are sometimes mistaken for diphtheria, but there is, I think, a difference even when looked at from a clinical point of view, without reference to the bacteriological test. The patient with follicular tonsillitis is not nearly so ill as one suffering from even mild diphtheria. There is seldom albumen present in the urine, and the exudation rapidly clears off under the administration of chlorate of potash and iron, and the use of disinfecting spray or gargles. And yet, I suppose, there must be some connection between them, for I have several times seen one mistaken for the other. One thing is certain, I think, these cases are very infectious, and I invariably isolate them. They were much more prevalent in the school some years ago than they are now, owing partly, I think, to the improvements that have been made in the drainage of the College houses.

The third form of sore throat I have observed is purely catarrhal. The symptoms are, intense redness of the fauces and pharynx, tonsils and palate, but with very little if any swelling; there is dryness of the mucous membrane, pain in swallowing, and fever. This is also infectious, but not so much so as follicular tonsillitis. There are also affections of the upper air passages, the nose, larynx, and trachea, acute and inflammatory in character, but I have never seen them assume an epidemic form except when complicated with influenza.

With regard to **scarlet fever,** I think the College has been wonderfully free from invasion from this disease, about which there exists so much apprehension in the public mind. I have never seen what I could call an epidemic of scarlet fever in the school. The only approach to it has been during the first term of the present year, when suddenly six cases developed in one house within a week, and a seventh in another house two days later. All these occurred in the junior school, but no further case appeared. Last summer a similar outbreak took place. Two cases occurred in one house, and a third in another, all in the upper school. There were no more. I need not say the cases were instantly isolated, and thorough disinfection of dormitories and infected studies was carried out at once. Formidable a disease as scarlet fever is when the type is severe in character, it is probably more under control than any of the other infectious fevers which threaten schools. I may add as a matter of fact that there have been only fifteen cases of scarlet fever in Clifton College among the boarders (for I have no medical charge of the Town boys) during the fifteen years I have been physician to the school. This leads me to say that notwithstanding the disparaging remarks I have read occasionally as to the inefficiency of sulphurous acid fumigation, I still believe in it, and always adopt it, combining with it the washing of floors, walls, and woodwork with carbolic solution. At any rate this is the readiest and most convenient way of disinfection I know of, and I have always found it successful in stopping the spread of scarlet fever in the school. With reference to the period of incubation of scarlet fever, I think it seldom exceeds eight days, but the Medical Officers of Schools Association puts it down at fourteen. I think there is an unnecessary scare when scarlet fever cases occur in a large school. I hardly think it is justifiable unless the type of the disease be malignant. If this form were to appear it might become the duty of the school doctor to advise the closing of the house in which it appeared, or even the entire school; but with the type we usually see, such a course is not only unnecessary but mischievous, and should only be adopted in the very last extremity.

Although we have never in my experience had to deal with an extensive epidemic of scarlet fever; it is a very different matter with regard to **measles.** We have had no outbreak of measles since 1889, but we may have one at any time, as I know there are many boys now in the school unprotected by previous attacks. Most large public schools experience an epidemic of measles about once in five years. When it comes we generally have about 100 cases. It is impossible to stop its progress once it gets a footing, for it is very infectious in its early stages, even before the rash appears, and fresh outbursts take place once in fourteen days, until every unprotected boy has been attacked. Disinfection seems quite useless. Measles has given me a great deal more trouble and anxiety than scarlet fever, on account of the complications which arise, especially bronchitis, followed by asthma and acute purulent inflammation of the middle ear.

I have never seen a case of **small-pox** in the school in the fifteen years I have been attached to it. The present Head Master introduced an excellent system when he was appointed nearly seven years ago, by which every new boy coming to the school is re-vaccinated in his second term, with the consent of his parents, if he has not been re-vaccinated successfully before. I think, therefore, there are now very few unprotected boys among the boarders.

**Chicken pox** is often met with, and most unprotected boys catch it if it enters a house. Most people think very lightly of it, but I have seen some severe cases, much resembling modified small-pox. It is a provoking disease to parents, who are worried by the necessary period of twenty-one days quarantine for the other young members of their family who have been exposed to the infection.

I have only seen one case of **diphtheria** during the fifteen years. The boy brought it at the beginning of a term from his home, where a few days after five other cases occurred among members of his family. It was before the days of antitoxin, and unfortunately, to my great regret, the boy died, not from extension of the disease to the larynx, but from cardiac failure.

Although I have not classed **typhoid fever** as one of the infectious diseases of schools, I may say we had one outbreak of it in 1884. There were, I think, three cases from the same house, and one death on the tenth day of the disease. It was this occurrence which led me to put before the Head Master and the Council the necessity of a thorough inspection of every Master's house and of the whole of the school premises: this was done, and the result has been the reconstruction of the drainage of every house in the school and of the sanatorium, carried out on the system of Mr. R. Field, who, I think, is the leader among sanitary engineers in the kingdom. Whether the cases of typhoid at this time were the result of faulty drainage in that particular house I cannot say, but at any rate we have had only one case of typhoid since, and the cases of infectious sore throat which used to be rather prevalent have been very infrequent since the improvements were made.

**German measles**, or as it is now called by the College of Physicians, **rubella**, is quite as difficult to stamp out when it obtains a footing in a school as the ordinary measles. As I have on a former occasion made this disease the subject of a paper published in the *Journal* of our Society, I will not say more than this, that the epidemic on that occasion assumed features which were clinically almost indistinguishable from scarlet fever, and were a puzzle not only to myself but to some of my most experienced medical friends. I must refer you to the paper in question, published in our *Journal* for March, 1892.

With reference to **whooping cough** I have very little to say. I seldom see a case of it except among the little boys in the preparatory school, and owing to the long duration of the malady and protracted recovery from it, parents as a rule prefer to take the boys to their homes.

I remember hearing an excellent paper on this disease read in the section for children's diseases at our meeting of the British Medical Association in Bristol in 1894, by a physician from Edinburgh, in which he strongly advocated the necessity of including whooping cough among the other infectious diseases mentioned in the Notification Act. Seeing that whooping cough causes a greater mortality than any of the other diseases of childhood, there seemed to me to be fair grounds for advocating this proposition.

With regard to **mumps**, I think without exception it is the most vexatious and troublesome of all the infectious diseases which affect public schools; and its entrance is most difficult to prevent. During the autumn term of last year we had the most severe epidemic of mumps I have ever seen in the school. There were 110 cases, some of them very severe.

In this disease there is again some difference of opinion respecting the period of incubation and the duration of infectiousness after recovery. One distinguished school medical officer of my acquaintance, upon whose opinion I have generally been disposed to pin my faith, holds that boys may safely be sent back to school after an attack of mumps in sixteen days, provided no swelling of submaxillary or other glands remain. I think this period is too short, for in this epidemic I found that cases of orchitis occurred after the sixteenth day, no enlargement of glands being present. The committee of the Medical

Officers of Schools Association advise that four weeks should elapse before a boy who has had mumps should return to school. This, on the other hand, is very long, and very trying to parents who have to keep their boys at home doing nothing all the time. The truth probably lies between the two points, and therefore I think that the period of twenty-four days is a safe medium both for the length of incubation after exposure to infection, and for the duration of infectiousness in recovery from an attack. In this recent epidemic of mumps there were fourteen cases of metastasis to the testicle, a very large proportion. Some of them were very severe and painful, and attended with a high degree of fever. I have never seen any instance of atrophy of the testicle[1] nor any case of affection of the brain such as one sees occasionally recorded.

**Contagious Impetigo.**—This is the well-known football rash. The boys of different schools give it names according to their own lively imaginations, such as mud fever, college leprosy. In one school the boys, with an ingenious attempt to combine the disease with that part of the game where its home may be said to lie (the scrimmage), call it scrum-pox. It is known in Clifton as "The foot-and-mouth." There are some noticeable points about it. It is generally confined to the upper school. I have never seen a case in the junior school. It is almost exclusively confined to schools where the Rugby game is the rule; very few Association players suffer from it. Then the boys who are mostly affected are the forwards. This narrows down and localizes its *habitat* to the scrimmages, where the rubbing and scraping of heads and faces against rough and soiled jerseys, against the football itself, against muddy ground, and against each other are fertile places for the spread of this disease, provided only there be one boy in the scrimmage who has a small patch of impetigo on some part of his face or head. Mr. H. G. Armstrong, the medical officer of Wellington College, has investigated this subject very exhaustively, and has printed a paper which he read before the Medical Officers of Schools Association last year. He sent circulars to the different schools, and received replies

[1] Later examination showed that amongst the cases in which the testicle was affected wasting occurred in three.

from thirty-seven. He found that where the Association game is played, with two exceptions, the disease is unknown, and of fourteen schools where Rugby rules exist it is unknown in *four*, while at ten others it is of frequent occurrence.

The disease itself begins as a reddish spot, on which a vesicle forms, which bursts and forms a crust, under which there is a yellowish raw surface. It spreads not only from one boy to another by actual contact, but by auto-inoculation. I have never, I think, seen the disease upon any part of the body except the face, ears, behind the ears, and upper part of the neck.

Mr. Armstrong had the discharges from the sores submitted to bacteriological examination, under the direction of the Clinical Research Association. The following is an extract from Dr. Galloway's report[1]: "The investigations were conducted on the discharges and scabs and on inoculation directly made on culture media from the vesicles occurring on six patients affected. In all these cases there were obtained the staphylococcus pyogenes aureus, or albus, or both. Staphylococcus aureus seems to be the predominant organism. There seems, therefore," says Dr. Galloway, "no doubt that the pustular dermatitis from which the boys suffer is really an impetigo, but of a much more virulent form than the ordinary impetigo."

Dr. Walter Smith points out some of the recent advances in the etiology of diseases of the skin, one of them being the knowledge of the fact that "impetigo, boils, and carbuncles are invariably produced only under the influence of micro-organisms." He stated, as the result of investigation by Bockhardt and Garrè, that microphytes enter the skin generally through a breach of the surface, but they can "penetrate into the lymph-channels of the skin through the intact epidermis. . . . If the micrococci invade only the epidermis we have a superficial pustule—*i.e.* (*a*) impetigo; if the intruders find their way deeper down the hair-follicles and gland-ducts, we have . . . (*b*) a boil, phlegmon, or suppurative folliculitis, and a congeries of furuncular points form (*c*) a carbuncle."

If these statements be true, the prophylaxis of this troublesome disease is plain, and might be summed up in one word—

[1] Mr. Armstrong's paper. [2] *Dublin J. M. Sc.*, 1892, xciii. 1.

disinfection. The boys' clothing, especially the jerseys, worn in the matches must be boiled or disinfected with hot air, or still better steam; but the principal point is, by careful inspection of the forwards especially, to exclude from the game any boy who presents a suspicious patch on his face.

As to treatment, disinfecting lotions of carbolic or boracic acid are, I think, the best. But the best ointment I have ever used was suggested to me by my friend Dr. Harrison, consisting of equal parts of unguentum hydrargyri ammoniati and ungentum zinci oxidi. This generally cures the disease rapidly, unless in some cases where from constitutional causes the disease becomes tedious and intractable.

**Ringworm** is a very rare disease in public schools. I do not think I have seen more than half-a-dozen cases. Dr. Harrison has helped me on many occasions with his knowledge and experience of skin diseases, especially of ringworm.

I saw one outbreak of **ophthalmia** in the college, and it impressed me greatly by the rapidity with which it ran through a house full of boys in the junior school. It was not purulent, it was merely catarrhal.

I believe we do not consider **jaundice** an infectious disease, and yet we know there are reported occasionally outbreaks of this affection which it is difficult to account for on any other hypothesis. I met with one instance of this kind among the boys of the college; several cases occurred rapidly one after the other, principally from one house. I could not discover that it was due to any special food the boys had partaken of. The cases were not severe, and were probably catarrhal in character.

What can be done to prevent the introduction of infectious disease into a school?

The first necessity is to have a good set of printed rules, a copy of which is sent to the parents or guardian of each boy. As I have already said, sometimes they are disregarded or neglected; but as a rule parents do attend to them, and this is of the greatest assistance in preventing the introduction of infection, especially at the beginning of a term. Then a great deal can be done to mitigate and prevent the spread of infectious disease after its introduction by instant isolation, not only of the patient but of his bedding and clothes. That is easily effected in every public school which has a good sanatorium.

Again, sanitary inspections by the school doctor are of great importance. This has been done in Clifton College for many years at the beginning of each term, and a report sent to the Head Master.

Infection may enter a public school from many sources other than a faulty drainage system. It may arise from communication with a neighbouring town, which may be visited by the boys or by servants attached to the school. When an infectious epidemic exists in the neighbourhood of the school, the medical officer should be in frequent communication with the medical officer of health of the district. Dr. Davies most kindly supplies me with a map showing the places where the disease is most prevalent, and such locality can be placed out of bounds by order of the Head Master. Infectious illness occurring among masters' children or in the doctor's house must be dealt with stringently. Then infection may come from the laundry, from the dairy, or from contaminated water. Lately we have introduced into most of the houses the Pasteur-Chamberland filter, which I believe is the only filter of any value.

But, in spite of all that can be done and is done, the efforts of school doctors are often baffled, and the microbe, whatever it may be, defies their precautions and breaks its way through their strictest rules. Dr. Dukes, the able medical officer of Rugby School, suggests that nothing short of Government inspection of high-grade schools will make them approximate to an ideal condition, and that even that, without the annual publication of all cases of infectious disease occurring in them, would be insufficient. The publication of such a return would be productive of the most active reform in every public school in the kingdom, and of the severest competition for the highest state of sanitary perfection and the cleanest bill of health. Whether it would be possible or desirable to carry out such a scheme is another question.

DISCUSSION ON DR. FYFFE'S PAPER.

In the discussion which followed the reading of Dr. Fyffe's paper, Dr. A. J. HARRISON said he was inclined to regard six days as a complete cover for the period of incubation of scarlet fever. He considered the vapour of formaldehyde to be a better disinfectant than sulphurous acid, and that the cylinder of the Berkefeld filter is an improvement upon that of the Pasteur-Chamberland.—Dr. R. SHINGLETON SMITH said it was fortunate that Dr. Fyffe had been able to keep the careful records on which his present summary was founded. In reference to the contagiousness of the different kinds of sore throat and of common catarrh, Dr. G. F. Burder, a long time ago, had come to the conclusion[1] that common colds were in no way dependent on weather, climate, or period of the year.—Dr. J. O. SYMES remarked on the low infectivity of scarlet fever, contrasting this with rubella. He mentioned that in France children in the whooping stage of whooping-cough were regarded as non-infectious.—Mr. JAMES TAYLOR had once seen a case of orchitis due to mumps in an adult, who had a temperature of 105° and albumen in the urine. He suggested that perhaps metastasis to the kidney is possible.—Dr. E. MARKHAM SKERRITT said that Dr. Fyffe's experience of the slight infectiousness of scarlet fever in the early stage was in keeping with his own. So uniformly, in fact, did the disease fail to spread after early isolation of a case, that he had come to speak almost positively as to the immunity that would follow such isolation in any given instance. In this respect scarlet fever was a marked contrast to measles, the latter being highly infectious from the beginning, and hence almost always spreading in spite of early removal of the patient and careful disinfection.—Dr. PARKER called attention to the extreme difficulty of diagnosis between follicular tonsillitis and diphtheria in the early stages before albuminuria and paralysis occur. He had long looked in vain for some basis, but had not found one, which was thoroughly trustworthy, either in the cardiac or local symptoms, or even in the results of a single culture. He regarded the suggested possibility of the incubation of scarlet fever lasting more than seven days as most improbable, both from the recorded facts and from the analogy of other short-term fevers.—Dr. F. H. EDGEWORTH advocated the treatment of unilateral parenchymatous tonsillitis by large doses of quinine.—Mr. C. A. MORTON suggested that after many months examination should be made for possible atrophy of the testicles, the result of the orchitis complicating mumps.—Dr. FYFFE stated in reply that he regarded rubella as a much more infectious disease than scarlet fever, and remarked upon an epidemic of the former which had taken place in Clifton College, and the great difficulty of distinguishing the symptoms from those of scarlet fever. Enlargement of the post-cervical glands in cases of rubella is probably the most distinguishing feature. He emphasized his remarks respecting diagnosis between follicular tonsillitis and diphtheria in its early stage, and agreed that cultures should be repeated several times before absolute certainty is reached.

[1] *Bristol M.-Chir. J.*, 1884, ii. 217.

